# Polyglutamic Acid‐Based Elastic and Tough Adhesive Patch Promotes Tissue Regeneration through In Situ Macrophage Modulation

**DOI:** 10.1002/advs.202106115

**Published:** 2022-04-09

**Authors:** Qiuwen Zhu, Yi Hong, Yuxuan Huang, Yi Zhang, Chang Xie, Renjie Liang, Chenglin Li, Tao Zhang, Hongwei Wu, Jinchun Ye, Xianzhu Zhang, Shufang Zhang, Xiaohui Zou, Hongwei Ouyang

**Affiliations:** ^1^ Dr. Li Dak Sum & Yip Yio Chin Center for Stem Cells and Regenerative Medicine and Department of Orthopedic Surgery of the Second Affiliated Hospital Zhejiang University School of Medicine Hangzhou 310058 China; ^2^ Department of Sports Medicine Zhejiang University School of Medicine Hangzhou 310058 China; ^3^ Zhejiang University‐University of Edinburgh Institute Zhejiang University School of Medicine Key Laboratory of Tissue Engineering and Regenerative Medicine of Zhejiang Province Zhejiang University School of Medicine Hangzhou 310058 China; ^4^ China Orthopedic Regenerative Medicine Group (CORMed) Hangzhou 310058 China; ^5^ Department of Gynaecology The First Affiliated Hospital School of Medicine Zhejiang University Hangzhou 310003 China

**Keywords:** elastic and robust patch, macrophage modulation, poly(amino acid) materials, tissue regeneration, wet tissue adhesive

## Abstract

Adhesive patches are advanced but challenging alternatives to suture, especially in treating fragile internal organs. So far there is no suture‐free adhesive patch based on metabolizable poly(amino acid) materials with excellent mechanical strength as well as immunomodulation functionality. Here, a polyglutamic acid‐based elastic and tough adhesive patch modified by photosensitive groups on the surface to achieve robust light‐activated adhesion and sealing of flexible internal organs is explored. With the porous internal morphology and excellent biodegradability, the patches promote regeneration through a macrophage‐regulating microenvironment. Treated rabbits achieve rapid full‐thickness gastric regeneration with complete functional structure within 14 d, suggesting its robust tissue adhesion and repair‐promoting ability.

## Graphical abstract

An elastic and robust light‐controlled adhesive patch (LAP) is fabricated for suture‐free dynamic internal tissue adhesion. The adhesive patch plays a role in promoting tissue regeneration function beyond the simple wound sealing effect. Its appropriate degradation time and pro‐regenerative macrophage response generate a suitable immune microenvironment for in situ tissue repair and regeneration.

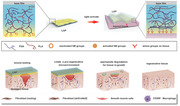

## 1 Introduction

Sutures and staples are the primary and gold‐standard methods for primary incisions closure. Nonabsorbable sutures with great tensile strength are preferable for those high‐tension tissues such as skeletal muscle and skin, whereas absorbable sutures are widely used in treating deeper wounds and most organs to facilitate further tissue self‐healing. However, as wound management plays an essential role in numerous medical practices, sutures are insufficient for complex clinical demands, especially for internal organ wounds. For example, sutures and staples may cause additional trauma to surrounding tissue and induce infection in sealing fragile organs such as lung,^[^ ^1^ ^]^ liver,^[^ ^1^, ^2^ ^]^ and spleen.^[^ ^3^ ^]^ Also, risks of leakages are high when applying sutures or staples to the closure of perforated tissues that demand very tight sealing, such as traumatized stomach^[^ ^4^ ^]^ and bladder.^[^ ^5^ ^]^ In these instances, developing a strategy to seal and repair the injured visceral tissue without causing further trauma to the organs is desirable.

Tissue sealants, which can bond strongly to and adapt to the dynamic biological tissues,^[^ ^6^ ^]^ have been a welcome substitute for sutures. However, tissue sealants in liquid form may not be suitable for visceral puncture wounds with large gaps, as the liquid may flow into the visceral cavity before solidification and cause internal adhesions or postoperative lumen blockage.^[^ ^7^ ^]^ As an alternative, suture‐free adhesive patches in solid form would work better on the organ with a functional luminal structure to prevent the infiltration of the cavity contents as well as withstand a certain tension from physiological or pathological activities of the viscera.^[^ ^8^ ^]^ Previous studies have reported a tough adhesive (TA)^[^ ^9^ ^]^ and a dry double‐sided tap (DST)^[^ ^10^ ^]^ to achieve efficient adhesion to the diverse wet surface for wound closure, both of which are in the form of tissue‐adhesive patches. Mechanical properties are prioritized when selecting materials for these adhesives, so mostly presented in the previous researches are synthetic polymers, such as polyacrylamide (PAM), polyacrylic acid (PAA), and polyethylene glycol, without which their elasticity will be inferior.^[^ ^11^ ^]^ However, with in‐depth research of biomaterials, the exploration of functional biomedical materials is more inclined to mimic the natural metabolites of the human body to resist the foreign‐body response and create a microenvironment conducive to tissue regeneration.^[^ ^12^ ^]^ In this context, biodegradable hydrogels based on poly(amino acid) have been considered as potential scaffolds for soft tissue implants because of their metabolizable hydrolysis units.^[^ ^12^, ^13^ ^]^ Polyglutamic acid (PGA) was chosen here to fabricate the matrix hydrogel of the patch because the internal electrostatic repulsion^[^ ^11^, ^14^ ^]^ and dense hydrogen bonding^[^ ^15^ ^]^ provided by the dense carboxyl groups inside may give the hydrogel both elastic and robust properties.

The strategy of tissue adhesion can be divided into two categories: physical adhesion and chemical adhesion. The physical adhesion mainly relies on hydrogen bonding, which is easily weakened by water and is not suitable for a humid internal physical environment. The chemical adhesion mechanism mainly includes carbodiimide reaction,^[^ ^9^ ^]^ photosensitive reaction,^[^ ^16^ ^]^ and cyanoacrylate reaction.^[^ ^17^ ^]^ Among them, only the photosensitive reaction can achieve controllable and strong tissue adhesion. Therefore, the light‐triggered adhesion might be the most suitable adhesion mechanism for suture‐free adhesive patches with operational controllability and convenience.

**Figure 1:**
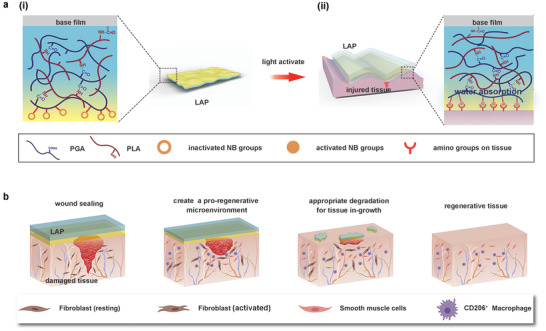
Mechanism and immune modulation of the LAP. a) (i) illustration of the design and the light‐activated tissue adhesion of the LAP. The fabricated LAP contains a PLLA base film (cyan square) and a matrix hydrogel which is crosslinked by two polypeptides (blue and red lines) whose surface (yellow part) is modified by photosensitive NB groups (orange hollow circle). (ii) After UV light illumination, the NB groups were activated (orange filled circle). When the LAP was pressed on the wet tissue, interfacial water was absorbed by matrix hydrogel (light blue region), and Schiff base was formed between the NB groups and the amino group on the tissue surface (red receptor). b) Schematic showing the wounds sealing of the LAP. After sealing the damaged tissue, the LAP was gradually degraded accompanied by the recruitment of the CD206^+^ macrophages to create a pro‐regenerative microenvironment for wound healing.

Here, we propose a biocompatible light‐controlled adhesive patch (LAP) designed for suture‐free closure and regeneration of visceral wounds with opening defects. The tissue adhesive patch consists of three parts, a highly absorbent matrix hydrogel made up of polypeptides, an adhesive surface modified with N‐(2‐aminoethyl)‐4‐(4‐(hydroxymethyl)‐2‐methoxy‐5‐nitrosophenoxy) butanamide (NB) groups, and a base film prepared by poly(l‐lactic acid) (PLLA) to enhance the mechanical strength of the patch (**Figure** **1**a). The NB is a high‐efficiency photosensitive group, which was reported in our previous work to achieve strongly adhesive hemostatic hydrogel,^[^ ^16^ ^]^ while the FDA‐approved polymer PLLA has been proven to have postoperative anti‐adhesion effects in vivo.^[^ ^18^ ^]^ After having the prepared LAP dried to a film, covered by nonstick paper and stored in a vacuum bag (Figure S1, Supporting Information), the LAP is easy to store and preserve, convenient to carry and use, simple and quick to cut into any shape before usage. The light‐activated LAP can rapidly and firmly seal the open wound of the multiple internal organs by simply pressing onto the defect site for 15 s. In vivo experiments suggested that the LAP has excellent biodegradability, and can provide an immune microenvironment that is conducive to tissue regeneration and angiogenesis (Figure 1b). The LAP proved to be competent for suture‐free wound closure and full‐thickness repair of the stomach in rabbit gastric perforation models. We anticipate that the progress achieved in the present study showcases the next‐generation adhesive patches with both mechanical properties and macrophage‐modulatory capabilities.

## 2 Results and Discussion

### 2.1 Optimization of Water Absorption and Mechanical Properties of LAP

**Figure 2:**
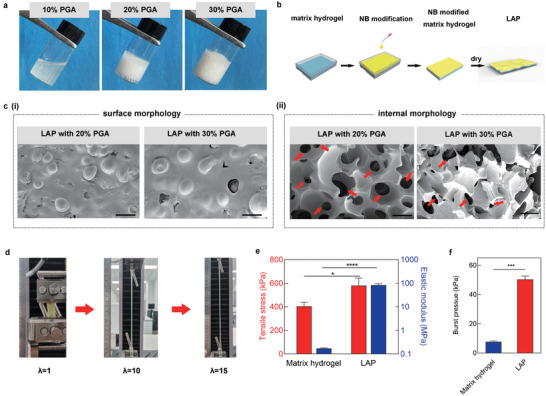
Microstructure and mechanical properties of LAP. a) Photographs of matrix hydrogel with different PGA concentrations. b) The fabrication process of the LAP. c) SEM images of (i) surface and (ii) cross section of LAP with different PGA concentrations. Red arrows indicate the internal pore of LAP (Scale bar: 500 µm). d) The matrix hydrogel exhibited an elongation up to 1500%. e) Tensile stress and elastic modulus of the matrix hydrogel and LAP. f) Burst pressure of the matrix hydrogel and LAP. Values represent the mean and the standard deviation (*n* = 3). *P*‐values were determined by Student's *t*‐test; *p < 0.05, ***p < 0.001, ***p < 0.0001.

Tissue wounds are usually covered by a large amount of blood and other liquids. These liquid molecules usually form barriers between the tissue surface and the adhesives to weaken the adhesion performance.^[^ ^19^ ^]^ Therefore, good water absorption property helps to eliminate the influence of liquid at the interface, allowing the patch to fully contact the tissue surface to achieve strong adhesion (Figure 1a). First, we optimize the selection of formulas with better water absorption properties by adjusting the concentration of PGA. The matrix hydrogel of LAP is based on polypeptides *γ*‐polyglutamic acid (PGA) and formed by coupling the carbonyl group in the PGA with the amine groups in another polypeptide poly‐l‐lysine (PLA). Matrix hydrogel at the concentration of 10%, 20%, and 30% (w/v) PGA was firstly synthesized under a certain feeding ratio of PGA: PLA. The PGA at the concentration of 10% cannot form a gel, while the PGA at the concentration of 20% formed a relatively loose hydrogel, and the PGA at the concentration of 30% formed a dense hydrogel (**Figure** **2**a). The resulting matrix hydrogel was then grafted with NB on the surface and dried at 65 °C to form LAP (Figure 2b). The surface and cross‐sectional structure of the dried samples with different amounts of PGA content were compared using scanning electron microscopy (SEM) analysis. The LAP with 20% and 30% concentration of PGA has a similar surface structure (Figure 2c and Figure S2a, Supporting Information). With the decrease of PGA concentration, the porosity of matrix hydrogel increases (Figure 2c and Figure S2b, Supporting Information). Although the water absorption ratio of LAP containing 20% PGA and 30% PGA were comparable after immersing in water for 10 min, the water absorption rate of the former (85%) at 15 s is significantly higher than that of the latter (57.5%) (Figure S3, Supporting Information). The instantaneous and rapid water absorption capacity of 20% PGA ensures that LAP can quickly absorb the moisture between the adhesive layer and the surface of the tissue.

Subsequently, we optimized the adhesion properties of LAP by adjusting the ratio of PLA. The optimized LAP had the highest tissue adhesion properties as assessed by measuring the coating adhesion strength. The coating adhesion strength of the LAP increased from 4.12 to 11.37 and 90.73 kPa with increasing feeding ratio of PGA:PLA until the ratio is 5:1 (mol:mol), where the LAP exhibited the highest coating adhesion strength of 143.1 kPa (Figure S4, Supporting Information). The prepared optimal LAP with 20% PGA concentration at the PGA:PLA ratio of 5:1 can favor the adhesive patch application because of its rapid water removal and tight tissue interaction.

As a suture‐free adhesive patch for visceral wounds, the LAP needs to bear the traction force while adhering to the internal organs, which requires a certain degree of elasticity. The fabricated matrix hydrogel without the base film exhibited an elongation up to 1500% (Figure 2d) at a stretching speed of 50 mm min^−1^. The ultimate elasticity of the matrix hydrogel may be due to the high‐density hydrogen bonds formed between a large number of carboxyl residues on PGA.^[^ ^15^ ^]^ To limit the deformation of LAP against the traction force on wound sealing, we further chose a hydrophobic base film prepared by poly(l‐lactic acid) (PLLA) to enhance the mechanical strength of the patch. The base film was attached to the back of the matrix hydrogel by in situ gel forming on the base film in the mold, where the free carboxyl groups on the base film will form chemical bonds with the free amino groups in the matrix hydrogel (Figure S5, Supporting Information). When the prepared LAP was dried, the hydrogen bonds formed between the interface, tightly connecting the two sections. And thus, the PLLA base film was attached to the matrix hydrogel by hydrogen bonds as well as amide linkage between carboxy end‐capping in PLLA and free amine groups in PLA (Figures 1a and 2b). The mechanical properties of LAP and matrix hydrogel were characterized by performing tensile and burst pressure tests (Figure 2e,f). Tensile tests showed that the tensile strength of LAP was 580.63 kPa, around 1.5 times higher than the bare matrix hydrogel (403.93 kPa) (Figure 2e). This value exceeds the maximum tension that the smooth muscle of the porcine stomach could bear (10.4 N cm^−2^, ≈104 kPa).^[^ ^20^ ^]^ Meanwhile, the elastic modulus of the LAP was sharply increased to 84 MPa from the origin 0.17 MPa of bare matrix hydrogel (Figure 2e). After the hydrophobic base film is broken, LAP can still withstand the stress of 150 kPa (Figure S6, Supporting Information), around three times higher than the previously reported protein‐based sealant (52.6 kPa) for lungs and arteries and the FDA‐approved surgical lung sealant Progel (42.1 kPa).^[^ ^21^ ^]^ The mechanical strength improvement of the adhesive patches was further determined by the burst pressure test (Figure 2f). The final LAP showed burst pressure of 50.48 kPa, nearly seven times higher than that of the matrix hydrogel without PLLA base film (7.7 kPa), but either are enough to withstand the maximum pressure exerted on the stomach during pathological gastric dilation (≈2.94 kPa).^[^ ^8^ ^]^ These results indicated the tremendous potential of the flexible and robust LAP in the internal organ repair.

### 2.2 In Vitro and Ex Vivo Light‐Activated Adhesion and Sealing Testing on LAP

**Figure 3:**
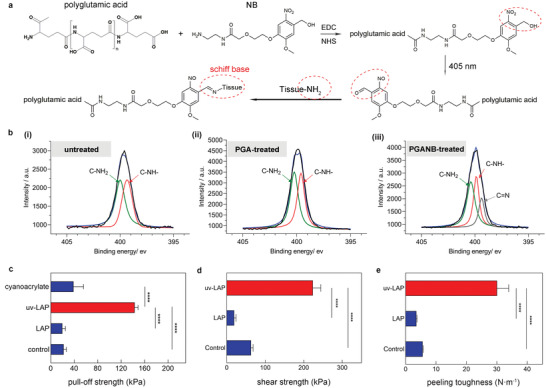
Light‐activated wet tissue adhesion of LAP. a) The chemical constituent and structure of adhesive layer and its reaction with tissue upon UV light irradiation. The *o*‐nitrobenzene groups were converted to *o*‐nitrosobenzaldehyde groups upon 405 nm irradiation and then formed Schiff base with amino groups on the tissue surface. b) X‐ray photon spectroscopy (XPS) assessment of porcine sausage casings. X‐ray photon spectrum showed (i) the untreated sausage casing, (ii) PGA‐treated sausage casing, and (iii) UV‐irradiated PGA‐NB treated sausage casing. The bond energies of C—NH bonds and C—NH_2_ bonds of untreated sausage casing were located at 399.3 and 400 eV. The bond energies of C—NH bonds and C—NH_2_ bonds of PGA‐treated sausage casing were located at 399.6 and 400.3 eV. The bond energies of C—NH bonds and C—NH_2_ bonds of the PGANB‐treated group were shifted to 399.9 and 400.4 eV, respectively, with a C═N bond peak located at 399.4 eV. c) Pull‐off tests of light‐activated adhesion strength between wet porcine muscle and LAP. d) Lap shear tests of light‐activated adhesion strength between wet porcine muscle and LAP. e) Peeling tests of light‐activated adhesion strength between wet porcine muscle and LAP. Control, unmodified matrix hydrogel attached by PLLA base film; LAP, nontriggered LAP; uv‐LAP, UV light‐triggered LAP. Values represent the mean and the standard deviation (*n* = 3). *P*‐values were determined by one‐way ANOVA; ****p < 0.0001.

Wound sealing in minimally invasive surgery is usually performed through a narrow catheter, so the adhesive film requires controllable adhesion to avoid either self‐adhesion or abnormally adhesion to the catheter. To achieve controllable robust adhesion to wet tissue, the LAP was designed to respond to UV light. The photosensitive NB group was conjugated to the carboxyl groups of the PGA by a condensation reaction. Under UV light (405 nm) irradiation, o‐nitrobenzene groups of NB converted to *o*‐nitrosobenzeldehyde groups and further reacted with amino groups on the tissue surface (**Figure** **3**a). The modification and light‐induced conformational change of the adhesive groups were monitored by Fourier transform infrared (FTIR) spectroscopy. Compared with nonmodified matrix hydrogel (Control), the UV light‐irradiated LAP (uv‐LAP) surface showed the disappearance of the peak of stretching vibration of —NO_2_ at 1520 and 1327 cm^−1^ accompanied by the presence of the peak of C═O of the aldehyde group at 1702 cm^−1^ in the uv‐LAP^[^ ^22^ ^]^ (Figure S7, Supporting Information). The interaction of o‐nitrosobenzeldehyde groups with tissue surface was further determined by X‐ray photoelectron spectroscopy (XPS). The appearance of C═N bonds and the consumption of C—NH_2_ on the tissue surface compared to the origin states supported our hypothesis that Schiff bases were formed between uv‐LAP and tissue (Figure 3b). As demonstrated in Movie S1 (Supporting Information), the irradiated LAP can firmly adhere to the wet pork surface, while the nonilluminated LAP has no adhesion ability. In addition, the moistened LAP can curl freely, and adhere to neither LAP itself nor other instruments abnormally, providing the possibility for minimally invasive sealing surgery.

We measured the light‐triggered adhesive capability of the adhesive patches to wet tissue by different types of adhesion tests on three groups, i.e., patches without NB modification (Control), LAP before irradiation (LAP), and the UV light irradiated LAP (uv‐LAP). Muscle tissue was chosen as the model tissue for adhesion detection, owing to its wide distribution in vivo. Coating adhesion test based on the ASTM D 4541 standard was performed to determine the “pull‐off” strength that the LAP can bear before detached. After short irradiation (405 nm, 30 mW cm^−2^, 15 s), uv‐LAP showed a markedly robust connection with wet muscle tissue with the peak pull‐off strength of 149.4 kPa, significantly higher than that of untriggered LAP (19.7 kPa) and nearly four times as high as that of the commercially available cyanoacrylate‐based glue (38.6 kPa) (Figure 3c and Figure S8a, Supporting Information). We found that the failure of the LAP during the coating adhesion test occurred at the interface between the base film and matrix hydrogel, given that the complete matrix hydrogel was still on the tissue after the test (Figure S9a, Supporting Information). We then removed the base film and re‐tested the coating adhesion between uv‐LAP and muscle tissue. As shown in Figure S9b (Supporting Information), cohesion failure occurred in the matrix hydrogel, leaving the residual of the adhesion layer on the tissue still intact. These results suggested that the adhesive strength between the adhesive patches and tissue surface exceed the cohesive strength of the matrix hydrogel itself. Lap shear test and peeling test were further used to determine the light‐induced adhesive capability of the LAP. Both the peeling strength and the shear strength of the uv‐LAP were improved by ten times compared to LAP before irradiation, up to 30 N m^−1^ (Figure 3d and Figure S8b, Supporting Information) and 223.8 kPa (Figure 3e and Figure S8c, Supporting Information), respectively. The result was consistent with the previous spectroscopic data (Figure 3b and Figure S7, Supporting Information), indicating that the activated photosensitive groups can form covalent bonds with a large number of amino groups on the tissue surface.

**Figure 4:**
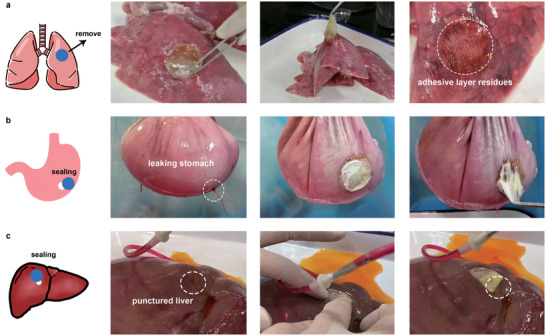
Adhesion and sealing performance of the LAP on various soft tissues. a) LAP robustly adhered to the ex vivo porcine lung and lifted the tissue while it was being removed. There were obvious adhesive layer residues on the surface of the porcine lung after detachment. b) Tough sealing of a leaking ex vivo porcine stomach by LAP. c) Fast sealing of a punctured ex vivo porcine liver by LAP.

To further investigate whether the robust adhesion capability of LAP can be applied to a variety of wet and dynamic tissues, we designed a series of proof‐of‐principle tests on ex vivo porcine organs. The LAP was first irradiated and then pressed on the surface of the moist porcine lung. Adhesion strength was assessed by lifting the tissue with the adhesive patch. As shown in Movie S2 (Supporting Information), the LAP with a diameter of 3 mm can completely lift a porcine lung weighing 500 g and maintain it for a while. There remains an adhesive layer on the surface of the porcine lung from which the patch is removed (**Figure** **4**a), indicating that the LAP had robust adhesion on the lung surface. Then we further explored the sealing potential of LAP as an adhesive patch for open wounds on soft tissues. A 5 mm hole was created on the porcine stomach filled with water. Obvious water jets spraying from the defect were observed. After applying the irradiated LAP to the notch, the wound was firmly sealed without leakage regardless of internal water pressure (Figure 4b and Movie S3, Supporting Information). Similarly, we created a 5 mm diameter puncture wound on the liver. By placing a water‐filled tube inside the puncture, we simulated bile leakage at the site of liver trauma. The activated LAP fitted well on the irregular liver surface and sealed the wound. The liquid that originally flowed out from the liver puncture lesion stopped. Instead, it flowed out from the backside (Figure 4c and Movie S4, Supporting Information). Such robust adhesion and wound sealing capability suggested the potential of LAP to treat diverse damaged tissues in vivo.

### 2.3 Biocompatibility and In Situ Immune Modulation of the LAP

**Figure 5:**
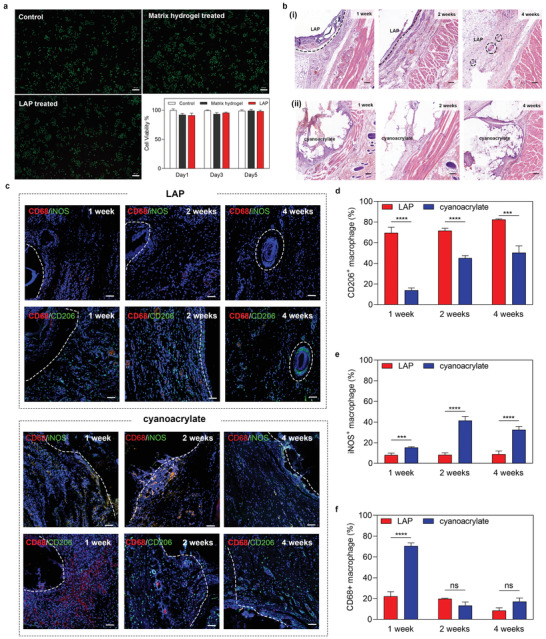
Biocompatibility and in situ immune modulation of the LAP. a) Live‐Dead staining of L929 cells treated by different medium for 24 h (*n* = 5) and the viability of L929 cells treated with different medium for 1, 3, and 5 d (scale bar: 200 µm). b) H&E staining of subcutaneously implanted (i) LAP or (ii) cyanoacrylate at 1, 2, or 4 weeks after implantation, respectively. The dotted line indicates the boundary between LAP and tissue (scale bar: 200 µm). c) Confocal imaging of immunofluorescence‐stained LAP or cyanoacrylate implant at 1, 2, and 4 weeks post‐subcutaneous implantation. Cell nuclei are stained with DAPI (blue). Green fluorescence corresponds to the expression of iNOS^+^ macrophages (up) or CD206^+^ macrophages (down). Red fluorescence corresponds to the expression of CD68^+^ macrophages. White dashed line indicates the boundary of the implanted sample (scale bar: 50 µm). d–f) Quantitative study of CD206^+^ macrophages, iNOS^+^ macrophages, and CD68^+^ macrophages at 1, 2, and 4 weeks post‐subcutaneous implantation, respectively. Values represent the mean and the standard deviation (*n* = 3). *P*‐values were determined by Student's *t*‐test; ***p < 0.001, ****p < 0.0001, ns: nonsignificant differences.

A competitive study was performed on the L929 fibroblast cells to evaluate the in vitro biocompatibility of the LAP by using cell counting kit‐8 (CCK‐8) and live/dead assay kit. The L929 cells treated by LAP conditioned medium showed comparable cytotoxicity to that of the control medium after incubation for the same period. The microscopic image also revealed that a majority of the fluorescence signal was living cells. On the contrary, the signal of dead cells was ignorable after short‐term incubation (24 h) (**Figure** **5**a).

Then we implanted the LAP subcutaneously in rats and collected samples for further assessment to explore the biodegradability of the materials and the microenvironment it generated in situ. Cyanoacrylate was selected as the negative control, because its internal use would induce an inflammatory response, delayed healing, and necrosis.^[^ ^23^ ^]^ The size of the implantation was calculated at different time points (1st, 3rd, 5th, 7th, 14th, 21st day) (Figure S10a, Supporting Information). The size of LAP increased sharply on the first day after implantation because of initial osmotic swelling, then gradually decreased within four weeks as the LAP degrade. The proportion of implanted size remaining after one week was 61.6%, after two weeks this had reduced to 40.8%, and only 18.7% remained after four weeks (Figure S10b, Supporting Information). In contrast, very little change was observed after cyanoacrylate was implanted (Figure S11, Supporting Information). Pathological staining with hematoxylin and eosin (H&E) also revealed the degradation process of the LAP. Notably, the LAP was gradually biodegraded into small pieces by macrophages and non‐inflammatory dividing cells that grow into the matrix hydrogel (Figure 5b). The fast degradation time of the LAP is synchronized with the regeneration of visceral smooth muscle, which usually peaks during the second week after injury^[^ ^24^ ^]^ and is practically complete on the 20th day.^[^ ^25^ ^]^ The match between material degradation and natural regeneration would greatly benefit functional tissue ingrowth. Conversely, if foreign matters like cyanoacrylate are left in place for a long time, it is likely to leave unwanted scars instead of forming a functional tissue.^[^ ^26^ ^]^ Based on these results, we speculate that the biocompatible glutamic acid‐based LAP can promote the rapid regeneration of the vesicle trauma.

Biomaterials and their degradation products generate a tissue microenvironment that serves an essential role in wound healing and tissue regeneration. Adhesive patches designed for tissue regeneration should eliminate the foreign body response (FBR) and provide a pro‐regenerative tissue environment, which is usually characterized by specific macrophage responses and phenotype.^[^ ^27^ ^]^ Among them, high expression of CD206 is considered to inhibit inflammation and promote tissue regeneration, while high expression of iNOS is considered to promote inflammation.^[^ ^28^ ^]^ Immunofluorescence staining with M0 (CD68^+^), M1 (iNOS^+^ CD68^+^), and M2 (CD206^+^) macrophage marker showed the dramatic enrichment of CD206^+^ macrophage during the whole degradation process of the LAP (Figure 5c). The M2 macrophages accounted for 69.58% of the total number of macrophages recruited by LAP in the first week, and increased to 71.77% in the second week, then reached 82.57% in the fourth week (Figure 5d). Accompanying the recruitment of M2 macrophages is the formation of new blood vessels for tissue regeneration, which is indicated by the abundant expression of CD31^+^ cells (Figure S12, Supporting Information). On the contrary, the slow‐degrading cyanoacrylate recruited 86.1% inflammatory (M0 and M1) macrophages in the first week. In the fourth week, M0 and M1 macrophages still accounts for 49.67% of all, indicating the severe inflammatory response and delayed healing of the wounds (Figure 5c–f). These results proved that LAP and its degradation products have excellent biocompatibility and generate a suitable immune microenvironment in situ for tissue repair and regeneration (Figure 1b).

### 2.4 Rapid Full‐Thickness Repair of Gastric Puncture Injury of the LAP

**Figure 6:**
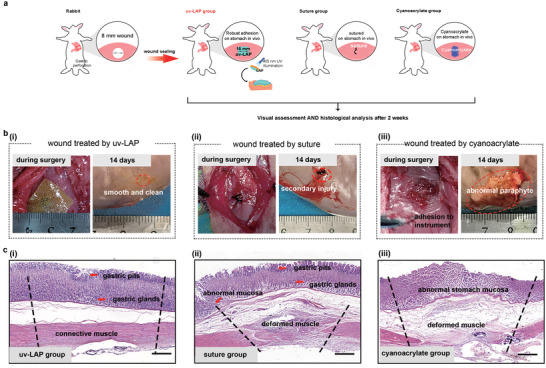
Sealing and regeneration of the gastric perforation by LAP. a) Schematic illustrations for experiments performed on punctured stomach (gastric perforation diameter is 8–10 mm). b) Rabbit's gastric perforation treated with (i) uv‐LAP, (ii) suture, or (iii) cyanoacrylate intraoperatively and after 14 d, respectively (*n* = 6). c) Microscopic images of H&E staining. The images showed the histological analysis of whole layer repair of the stomach treated with (i) uv‐LAP, (ii) suture, or (iii) cyanoacrylate. The dotted line represented the damaged area (Scale bar: 400 µm).

The in vivo tissue repair and regeneration of LAP were assessed on the New Zealand white rabbits. A gastric perforation model with a diameter of 0.8 cm was created on the stomach of rabbits, and the light‐triggered uv‐LAP was attached to the injury site (**Figure** **6**a and Figure S13a, Supporting Information). The uv‐LAP formed robust adhesion on the dynamic tissue in situ and did not fall off even if there was tissue peristalsis and further surgical traction. The surgical sutures and commercial cyanoacrylate were also used as experimental surgical comparisons. Both control groups introduced extra mess and inconvenience. Suture needle puncture caused secondary injury to the stomach during the surgical process (Figure 6b). On the other hand, cyanoacrylate liquid spread to the surrounding tissues and caused unexpected adhesions upon contact with anything before solidification (Figure 6b). We assessed the stomach repair of gastric perforation at 14 d after surgery. The surface of the stomach treated by LAP was smooth. The defect site recovered well and was almost invisible (Figure 6b). Although the wound treated with sutures was closed, postoperative tissue adhesion existed in the surrounding, together with obvious secondary trauma caused by sutures. Compared with the LAP and suture groups, the wound treated by cyanoacrylate was pessimistic because of the abnormal proliferation and paraphyte on the stomach. The mortality rates of the rabbits treated by sutures and cyanoacrylates were both 66% due to the peritonitis, while all rabbits treated with LAP survived (Figure S13b,c, Supporting Information), suggesting its excellent biocompatibility and sealing capability in vivo.

The histological analysis of the stomach was examined by H&E staining. As shown in Figure 6c and Figure S14b (Supporting Information), the perforated stomach treated by LAP achieved complete tissue regeneration including connective muscle layer, gastric glands, and regular gastric pit, comparable to normal tissue (Figure S14a, Supporting Information). Besides, neither significant pathological changes nor inflammation responses were found in new tissue. In addition, a thin layer remaining on the surface of the stomach confirmed our hypothesis that the degradation of the patch can match the regeneration of vesicle smooth muscle (Figure 6b,c). On the other hand, suture group showed inferior repair due to the excessive stretching of the wound and the secondary trauma. Although the gastric glands and gastric pits have been restored, gastric smooth muscle grew aberrantly, facing a risk of gastric leakage (Figure S14c, Supporting Information). The stomach treated by cyanoacrylates is abnormal with apoptotic mucosal cells and muscle cells mainly because of the poor biocompatibility (Figure S14d, Supporting Information). Taken together, these results suggested that the glutamic acid‐based LAP could serve as a suture‐free robust adhesive patch for rapid vesicle tissue repair and regeneration.

## 3 Conclusion

Though sutures and staples define the traditional methods for wound closure, their limitation prompted the birth of next‐generation adhesive patches. With the vigorous exploration of biomaterials, patches have evolved to meet versatile needs of human body, reaching beyond simple tissue sealing. The interaction between tissues and materials is increasingly considered in the development of functional implant materials. However, in order to improve the toughness of the suture‐free patch, most patches are based on synthetic polymers, such as PEG, PAA, and PVA, whose metabolites are also foreign bodies to the human body. Researchers strived to find a bio‐mimetic material as a substitute for synthetic materials, so as to obtain better biocompatibility. Poly(amino acid) is one of the most promising materials due to its diverse selection, metabolizable degradation units, and controllable synthesis.

In this article, we first fabricated a polyglutamic acid‐based adhesive patch with light‐activated wet tissue surface adhesion capability. The tensile test and burst pressure test of the LAP illustrated that this adhesive patch exhibits high elasticity and robust strength beyond the natural tissue (porcine stomach) without adding any synthetic polymer. The LAP was grafted by light‐activated groups to achieve controllable and strong tissue adhesion, without self‐adhesion or adhering to the instrument intraoperatively. This feature makes LAP potentially suitable for minimally invasive surgery. The rapid water absorption capability of the polyglutamic acid hydrogel matrix^[^ ^29^ ^]^ enables the LAP to swiftly remove moisture between the tissue surface and the activated adhesive group. Therefore, close contact and subsequent reaction could be ensured. Through ex vivo tissue adhesion and open wounds sealing tests, we proved the on‐demand robust adhesion and firm wounds sealing of LAP on a variety of wet tissue surfaces.

The LAP can be biodegraded and can simultaneously create a microenvironment suitable for tissue regeneration through in‐situ immunomodulation. This remarkable function is probably due to its appropriate degradation rate and its amino acid degradation products that are completely metabolizable. Through subcutaneous implantation experiments, we found that the degradation rate of LAP matches the regeneration rate of smooth muscle. Therefore, it is speculated that the LAP is suitable for suture‐free repair of the fragile visceral organs with smooth muscle. In situ repair of rabbit's stomach perforation verified the suture‐free tissue repair by LAP. The light‐activated LAP quickly adheres to the surface of the gastric surface, sealing the wounds and preventing gastric juice leakage. During gradual degradation of LAP, a microenvironment for smooth muscle regeneration as well as new tissue ingrowth was generated. The hydrophobic back membrane of LAP can prevent the postoperative tissue adhesion. Within two weeks, a rapid full‐thickness repair of the gastric perforation was achieved.

In summary, we designed a polyglutamic acid‐based light‐activated adhesive patch with excellent mechanical properties and the ability to simultaneously generate an appropriate microenvironment for tissue regeneration by macrophage modulation. The mechanical performance and in situ immunomodulatory capability enable the state‐of‐the‐art adhesive technology to potentially forward from bench to bedside.

## 4 Experimental Section

### Preparation of the Matrix Hydrogel

The matrix hydrogel was in situ gelled on the base film in a glass mold. For base film synthesis, the PLLA (80 000 Da) dissolved in chloroform was evaporated on the bottom of a glass mold (10 mg PLLA cm^−2^) to form a transparent base film. For matrix hydrogel precursor preparation, the PGA (700 000 Da, Saien Biotechnologies) solution was mixed with PLA (3500 Da, Yiyang Biotechnologies) solution at room temperature for 10 min, followed by addition of N‐hydroxysuccinimide (NHS, Aladdin) and 1‐ethyl‐3‐(3‐dimethylaminopropyl) carbodiimide (EDC, Aladdin) powder. The precursor was then vigorously stirred for 2 min and immediately poured on the base film in the glass mold to form matrix hydrogel at 37 °C. The molecular ratio of PGA (carboxyl groups):PLA (amino groups):NHS:EDC was 1:0.2:0.25:0.25. The final precursor is composed of 30% PGA for high‐concentration matrix hydrogel, 20% PGA for medium‐concentration matrix hydrogel, and 10% PGA for low‐concentration matrix hydrogel.

To obtain the optimized ratio of matrix hydrogel, the matrix hydrogel of four different molecular proportions of PGA:PLA (8:1, 7:1, 6:1, 5:1, 4:1) were prepared and their coating adhesion strength was tested.

### Preparation of the LAP

To modify the matrix hydrogel with a photosensitive group, EDC and NHS were dissolved in deionized water and added onto the surface of matrix hydrogel at the concentration of 0.02 mmol cm^−2^. Then the photosensitive group N‐(2‐aminoethyl)‐4‐(4‐(hydroxymethyl)‐2‐methoxy‐5‐nitrosophenoxy) butanamide (NB) was dissolved in the DMSO and added into the mixture at the concentration of 4 µmol cm^−2^. The mixture was reacted on a horizontal shaker for 30 min in the dark, and then washed by deionized water for five times. The modified matrix hydrogel was dried in the oven for 6 h at 65 °C to obtain the final LAP with the thickness of 200 µm unless otherwise specified. The LAP was vacuum‐packed with a polyethylene‐coated paper as backing and stored in a dry place for further use. The control group (Control) used for test was prepared by drying the unmodified matrix hydrogel only.

### Characterization of the Matrix Hydrogel

For microstructure characterization, the surface and cross‐sectional morphology of matrix hydrogel was observed with scanning emission electron microscopy (FEI SIRION‐100) after fractured by liquid nitrogen.

For water absorption determination, the matrix hydrogel was immersed into the deionized water for different time and the water absorption ratio (%) of matrix hydrogel was calculated by (*W*_1_ − *W*_0_)/*W*_0_ × 100%, where *W*_1_ is the weight after water absorption and *W*_0_ is the original weight.

### Mechanical Strength Tests of the Matrix Hydrogel

To measure the tensile strength of the LAP, the samples were cut into the pieces of width 1 cm and length 2 cm and tested by the standard tensile test (MTS Criterion model C41.103Y) with a mechanical testing machine at a constant strain speed of 50 mm min^−1^. The force and the displacement were recorded. The tensile strength was calculated by dividing the maximum force by the cross‐sectional area.

The burst pressure test was carried out according to the method previously reported in the literature. Briefly, a clean casing was cut into a size of 4 × 4 cm and secured to the testing device, which was linked to a pressure meter. A 2 mm diameter hole was created in the center of the casing, and then the adhesive patch with or without PLLA base film was covered onto the defect. Constant pressure was applied to the defect site by a microflow control system (7 mL min^−1^) to record the pressure meter reading, and the maximum reading on the pressure meter before the adhesive ruptures was recorded as the burst pressure.

### Light‐Activated Adhesion Mechanism Tests

XPS (Thermo Scientific ESCALAB 250Xi) was performed to characterize the surface composition of the porcine skin sausage with an A1 K*α* source (1486.6 eV). The porcine skin sausage was treated with PGA solution and UV‐triggered PGA‐NB solution for 5 min, respectively, and then washed with deionized water three times. The standard carbon 1s peak at 284.8 eV was used for calibration.

The surface of the LAP before and after uv‐activation as well as the unmodified control group were measured and analyzed by Fourier transform infrared analysis (Themo Fisher Scientific LLC, Nicolet 6700).

### Light‐Activated Adhesion Capability Tests

A 2 cm × 2 cm fresh pork sample with a wet surface was prepared and immersed in the cold PBS. To prepare the testing sample, the dry adhesive film was activated by UV activation (405 nm) for 15 s. Immediately after activation, the adhesive film was pressed on the surface of pork for 15 s to ensure the contact of the film and pork. All mechanical tests on the sample were conducted 10 min after initial pressing to obtain a balance of the adhesive film in wet environments. Cyanoacrylate glues were used following the manufactory guidelines (508 medical adhesive, Guangzhou Baiyun Medical Adhesive Co., Ltd.).

For coating adhesion test, back layers of both the pork and the LAP were glued to the middle of one side of a glass with superglue, with the other side covered by plastic sheets for machine grip. To test “pull‐off” strength, reverse‐direction tension was applied on the free end of the plastic sheets using an INSTRON (model 5944, max load: 500 N) machine at a stable loading rate of 20 mm min^−1^. Meanwhile, the force and displacement were recorded.

For peeling test, back layers of both the pork and the LAP were glued to an end of 4 cm plastic sheets with superglue, leaving half of the sheets free for machine grip. To test peeling adhesion energy, reverse‐direction tension was applied on the free end of the plastic sheets with an INSTRON (model 5944, max load: 500 N) machine at a stable loading rate of 20 mm min^−1^. Meanwhile, the force and displacement were recorded. Peeling toughness was determined as two times the maximum force divided by the width.

For shear strength test, back layers of both the pork and the LAP were glued to one end of glass with superglue, leaving the other end of the glass free for machine grip. To test shear strength, reverse‐direction tension was applied on the free end of the glass with an INSTRON (model 5944, max load: 500 N) machine at a constant loading ratio of 20 mm min^−1^. Meanwhile, the force and displacement were recorded. Shear strength was determined as the maximum force divided by the adhesion area.

### Ex Vivo Adhesion and Sealing Test

To assess the adhesion properties of the adhesive on the pig lung, the prepared LAP was triggered by UV light for 15 s, and then the uv‐LAP was pressed onto the surface of the wet pig lung for 15 s. The adhesion strength was determined 5 min after initial pressing by lifting the pig lung with the LAP.

To assess the sealing properties of the film on the pig liver, a 10 mm puncture wound was created on the pig liver and a drainage tube was inserted into the wound. PBS solution (added with Alizarin Red) at a flow rate of 7 mL min^−1^ was injected by a microfluidic system to simulate the broken liver. Then the prepared LAP was triggered by UV light for 15 s, and then the uv‐LAP was pressed onto the surface of the puncture wound to monitor the sealing capability.

To assess the sealing properties of the film on the pig stomach, a 10 mm puncture wound was created on the pig stomach. 500 mL water was injected into the pig stomach to guarantee an obvious water jet squirting from the defect. The prepared LAP was triggered by UV light for 15 s, and then the uv‐LAP was pressed onto the surface of the puncture wound to monitor the sealing capability.

### In Vitro Biocompatibility Test

The samples with the hydrogel mass of 200 mg were sterilized by irradiated sterilization and incubated with 10 mL DMEM supplement with 10% FBS and 1% penicillin/streptomycin at 37 °C for 24 h to obtain a conditioned medium.

L929 fibroblast cells (L929, cell bank of the Chinese Academy of Science) were seeded in a six‐well plate (1 × 10^5^ cell per well, *n* = 3) and incubated with conditioned medium for 24 h at 37 °C. After incubation, each well was treated with a live/dead cytotoxicity kit and observed by a fluorescence microscope (Olympus). Next, L929 cells were seeded in a 96‐well plate (5 × 10^3^ cell per well, *n* = 5). Cells were incubated with conditioned medium for 24, 48, and 96 h at 37 °C. Cell viability was detected using Cell Counting Kit‐8 (CCK‐8) cell proliferation and cytotoxicity assay. Briefly, conditioned medium was replaced by 100 µL DEME containing 10% CCK‐8 and incubated for 4 h. The absorbance was determined at 450 nm. The cell viability was calculated by *A*/*A*_0_ × 100%, where *A*_0_ and *A* are the absorbance of the blank and treatment groups, respectively.

### In Vivo Biodegradability and Immune‐Modulation Capability Test

All animals were treated according to guidelines approved by the Zhejiang University Ethics Committee (ZJU20200131). The subcutaneous implantation experiment was carried out on male rats (200‐250g). The sterilized LAP was cut into a round piece with a diameter of 8 mm. one piece of adhesive film was embedded into a subcutaneous pocket on the back of a rat (*n* = 6) while 1 mL cyanoacrylate was also subcutaneously injected as a negative control. The rats were euthanized after 1, 3, 5, 7, 14, and 21 d and the implanted regions were harvested for degradability evaluation. The samples harvested at 7, 14, and 21 d were fixed in 10% formalin for 3 d for histology and H&E staining and immunofluorescence analysis.

### Rabbit Stomach Repair Experiment

All animals were treated according to guidelines approved by the Zhejiang University Ethics Committee (ZJU20200131). Male New Zealand white rabbits weighed about 2.5 kg (*n* = 6) were used for the experiment. The adhesive film was sterilized by gamma irradiation before usage. The rabbits fasted for 12 h before the operation. Briefly, the abdomen of the rabbit was opened and an 8 mm puncture hole was created on the stomach. The wound was closed by either adhesive film with a diameter of 1.5 cm or cyanoacrylate glue (508 medical adhesive, Guangzhou Baiyun Medical Adhesive Co., Ltd.). Suture performance was carried out as a control. The rabbits were treated with amoxicillin for 3 d, postoperatively. The rabbits were given saline and sucrose solution for 6 d and returned to their normal diet. The rabbits were sacrificed and the stomachs were harvested after 14 d. The repair of the stomach of all the groups was recorded by camera and determined by histology and H&E staining analysis.

## Conflict of Interest

The authors declare no conflict of interest.

## Supporting information

Supporting Information

Supplemental Movie 1

Supplemental Movie 2

Supplemental Movie 3

Supplemental Movie 4

## Data Availability

The data that support the findings of this study are available from the corresponding author upon reasonable request.
