# Supplementary material for: Polyglutamic Acid‐Based Elastic and Tough Adhesive Patch Promotes Tissue Regeneration through In Situ Macrophage Modulation
Source: Adv Sci (Weinh). 2022 Apr 9;9(17):2106115. doi: 10.1002/advs.202106115 (PMC9189670; doi:10.1002/advs.202106115)

ADVANCED SCIENCE
Open Access

Supporting Information

for *Adv. Sci.*, DOI 10.1002/advs.202106115

Polyglutamic Acid-Based Elastic and Tough Adhesive Patch Promotes Tissue Regeneration through In Situ Macrophage Modulation

Qiuwen Zhu, Yi Hong, Yuxuan Huang, Yi Zhang, Chang Xie, Renjie Liang, Chenglin Li, Tao Zhang, Hongwei Wu, Jinchun Ye, Xianzhu Zhang, Shufang Zhang, Xiaohui Zou and Hongwei Ouyang**

Supplementary Materials for

Polyglutamic acid-based elastic and tough adhesive patch promotes tissue regeneration through in situ macrophage-modulation

Qiuwen Zhu[1,2,3]‡, *Yi Hong*[1,2,3]‡, *Yuxuan Huang*[1,2,3], *Yi Zhang*[1,2,3], *Chang Xie*[1,2,3], *Renjie Liang*[1,2,3], *Chenglin Li*[1,2,3], *Tao Zhang*[1,2,3], *Hongwei Wu*[1,2,3], *Jinchun Ye*[1,2,3], *Xianzhu Zhang*[1,2,3], *Shufang Zhang*[1,2,3,5], *Xiaohui Zou*[1,3,4]*, *Hongwei Ouyang*[1,2,3,5]*

‡These authors contributed equally to this work.

Figure S1.
The final package of the LAP.

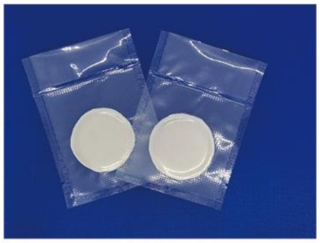

Figure S2.

The top view (a) and side view (b) of the matrix hydrogel, NB-modified matrix hydrogel and the dried LAP with different concentration of the PGA.

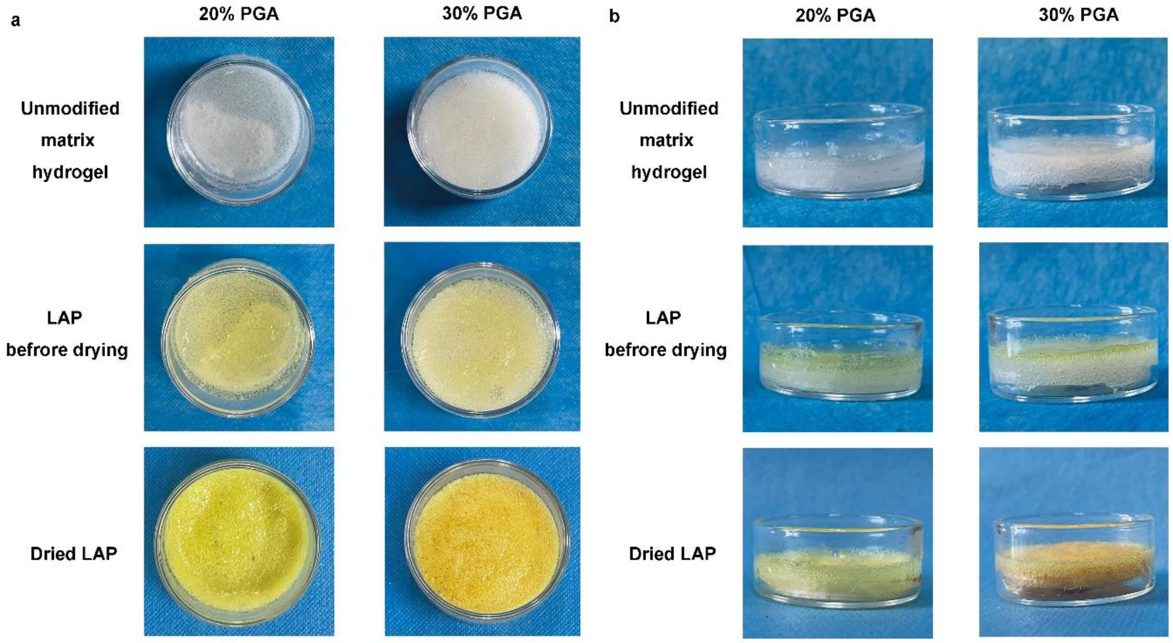

Figure S3.

Water absorption ratio of matrix hydrogel synthesized by different concentration of the PGA. Values represent the mean and the standard deviation (n=3). P value were determined by t-student test; ****p*< 0.001.

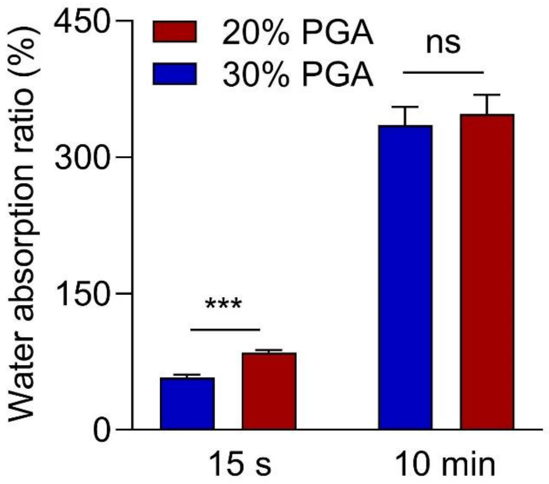

Figure S4.

Pull-off strength of LAP at different feeding ratio of the carboxyl group in PGA to the amino group in PLA (PGA: PLA). Values represent the mean and the standard deviation (n=3). P value were determined by one way ANOVA; ****$p < 0.0001$.

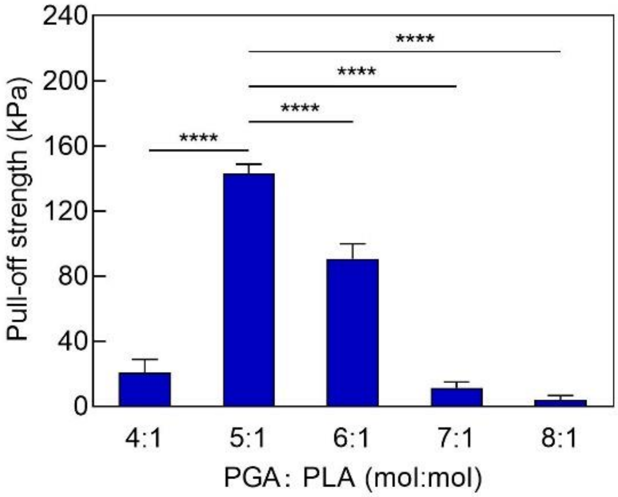

Figure S5.

Survey XPS spectra of PLA treated PLLA base film with (PLLA+PLA+END/NHS) or without (PLLA+PLA) coupling reagents, and untreated PLLA base film (PLLA). The survey XPS spectra of the PLLA+PLA+END/NHS clearly exhibit N1s peaks at a binding energy of 399.23eV, whereas the PLLA+PLA show a negligible N1s peaks and the PLLA does not have any N1s peaks in the corresponding energy range.

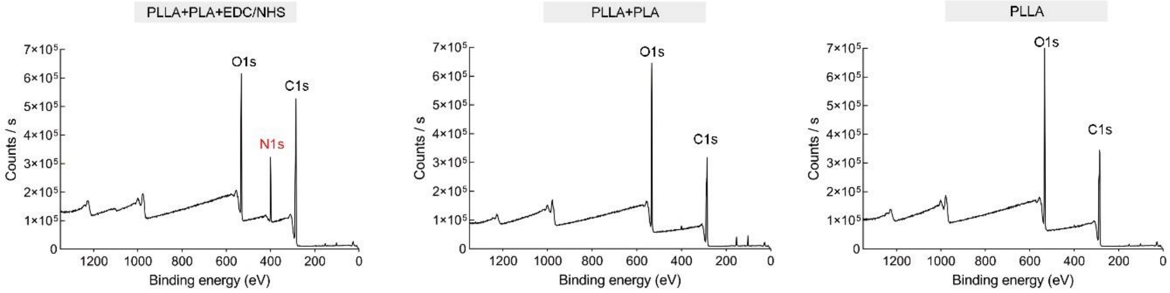

Figure S6.
The stress-strain curve of the matrix hydrogel and LAP.

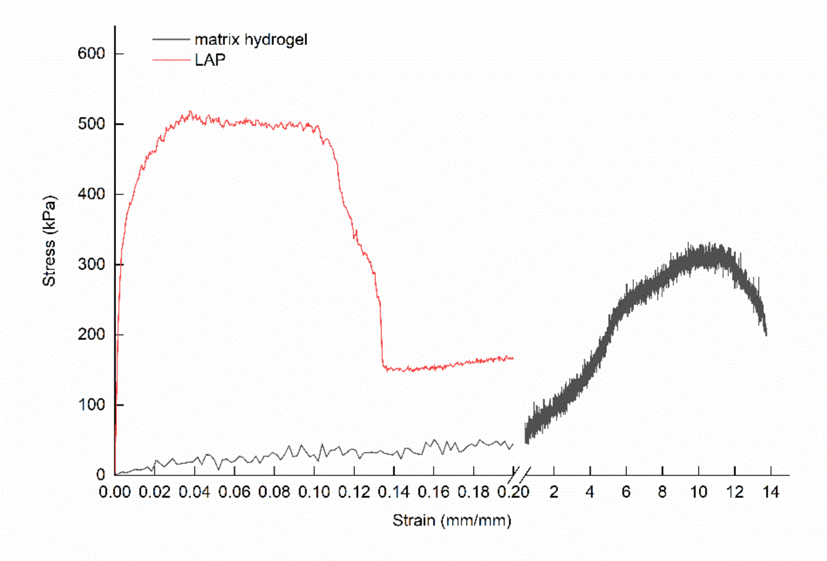

Figure S7.

FTIR spectra of the surface of the LAP before and after UV irradiation.

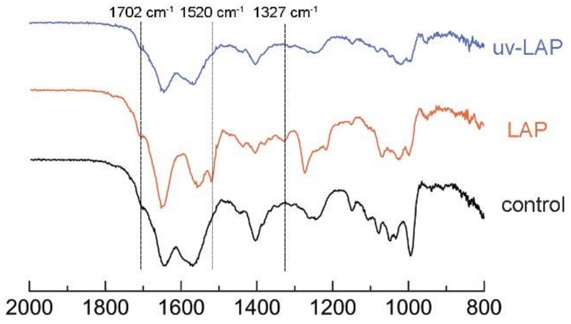

Figure S8.

Representative force curves of the LAP adhere to wet porcine muscle. a, pull-off strength vs. displacement curves for tensile tests of porcine muscle adhered by the LAP and cyanoacrylate. **b,** Force/width vs. displacement curves for 180-degree peeling tests of wet porcine muscle adhered by the LAP. **c,** Shear stress vs. displacement curves for lap-shear tests of wet porcine muscle adhered by the LAP.

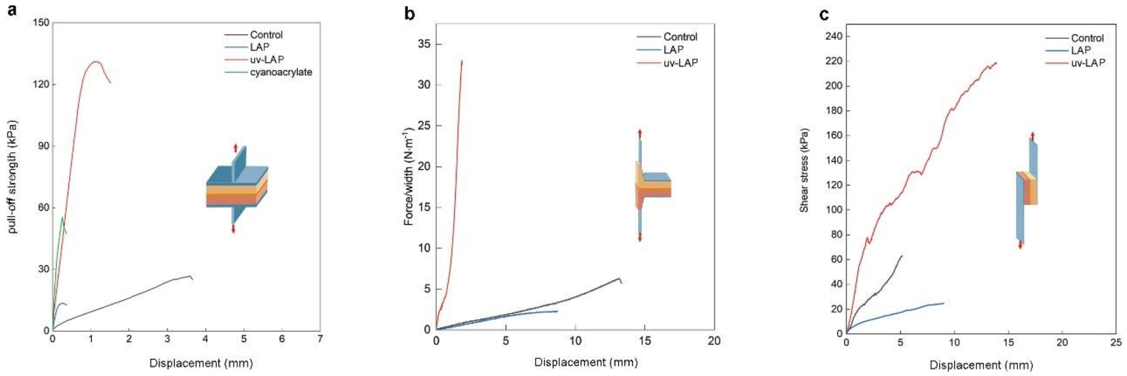

Figure S9.

Robust adhesion of the LAP on the surface of wet porcine muscle. a, The stretched LAP during the coating adhesion test and the broken LAP after the test. The broken LAP attached to the tissue surface. **b,** Coating adhesion test on NB-modified matrix hydrogel without base film. The fracture turned to be the internal connection of the matrix hydrogel.

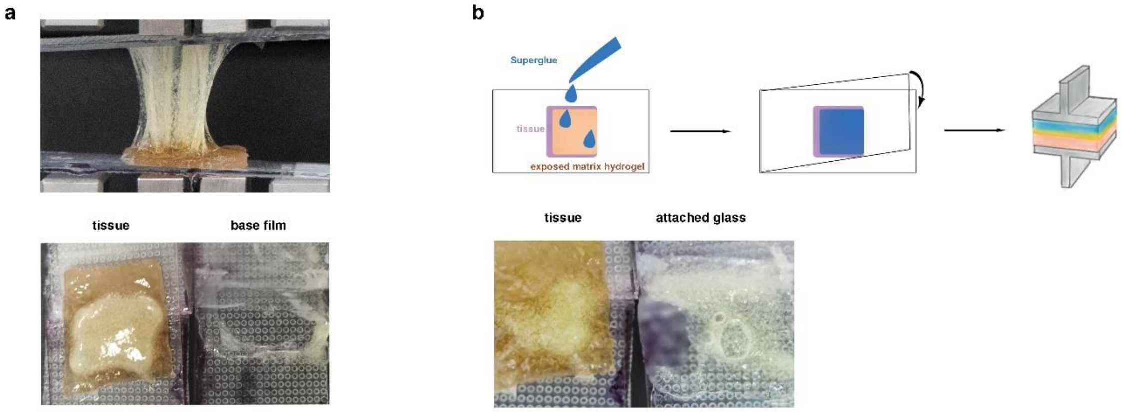

Figure S10.

Biodegradation testing of LAP after subcutaneous implantation. a, Schematic illustration of subcutaneous implantation with LAP. **b,** Degradation curve of LAP after implantation over time. (n=6)

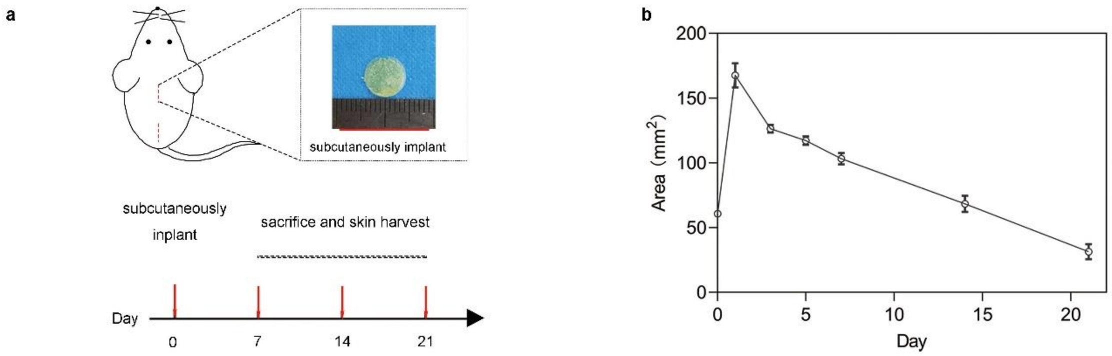

Figure S11.

Macroscopic view of LAP and cyanoacrylate implants at 1, 2 and 4 weeks post-implantation. (the red dotted circles point out the remaining implants)

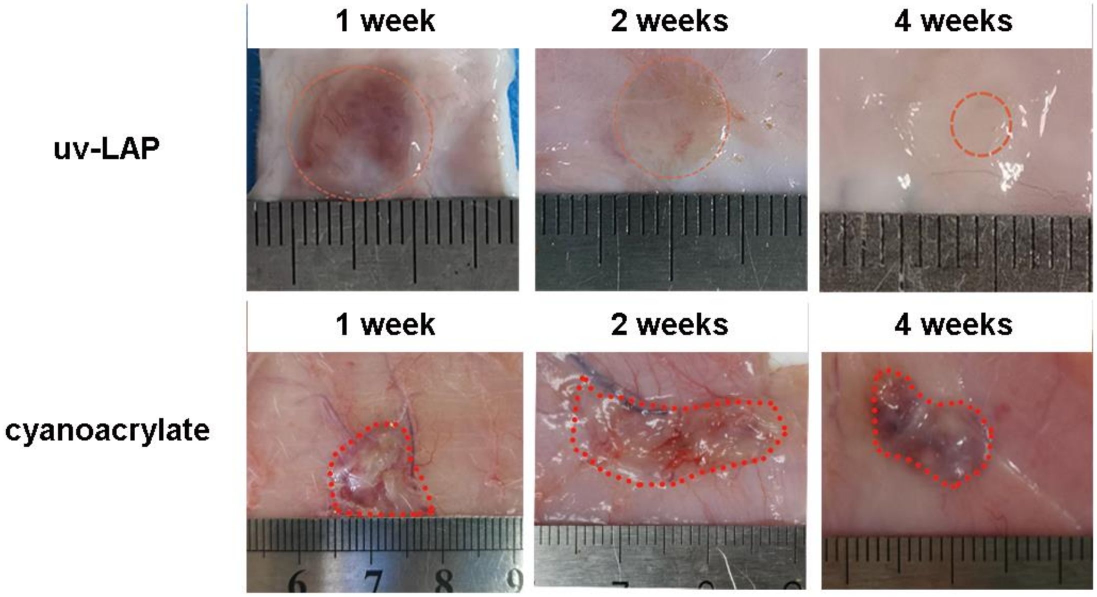

Figure S12.

Confocal imaging of immunofluorescence-stained LAP 1,2, and 4 weeks post subcutaneous implantation. Cell nuclei are stained with DAPI (blue). Red fluorescence corresponds to the expression of $CD31^+$ cells. White dashed line indicates the boundary of the implanted sample (scale bar 50μm).

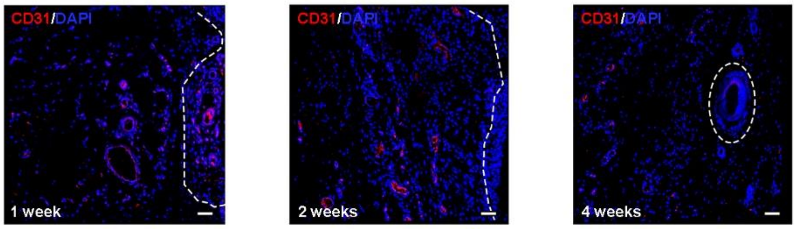

Figure S13.

In vivo application based on gastric perforation model. **a,** 8 mm diameter Gastric perforation model in rabbit was created by scissors. **b,** Survival of rabbits after gastric perforation rabbits treated with LAP, suture and cyanoacrylate. **c,** Anatomy of stomach from dead rabbits treated by suture and cyanoacrylate.

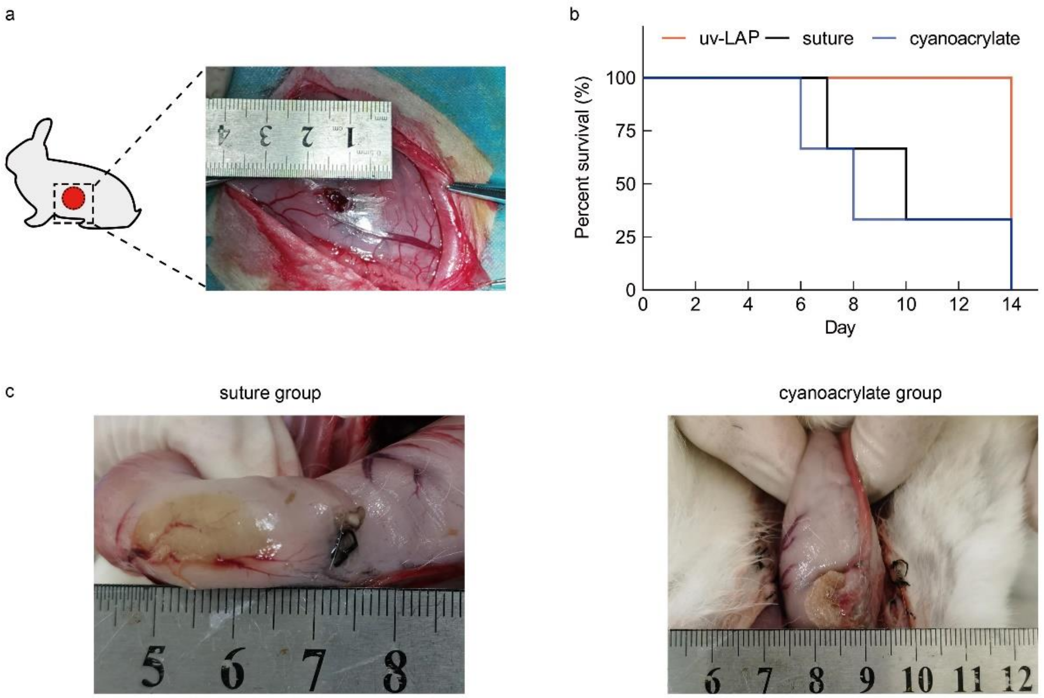

Figure S14.

Microscopic images of H&E staining. The images show the histological analysis of whole layer repair (up, scale bar 400 μm) and the regeneration of the muscularis and mucosa in the middle area of the gastric perforation (down, scale bar 100 μm) after treated by b) uv-LAP, c) suture, and d) cyanoacrylate. Unpuctured stomach is served as a positive control and dotted line represents the damaged area.

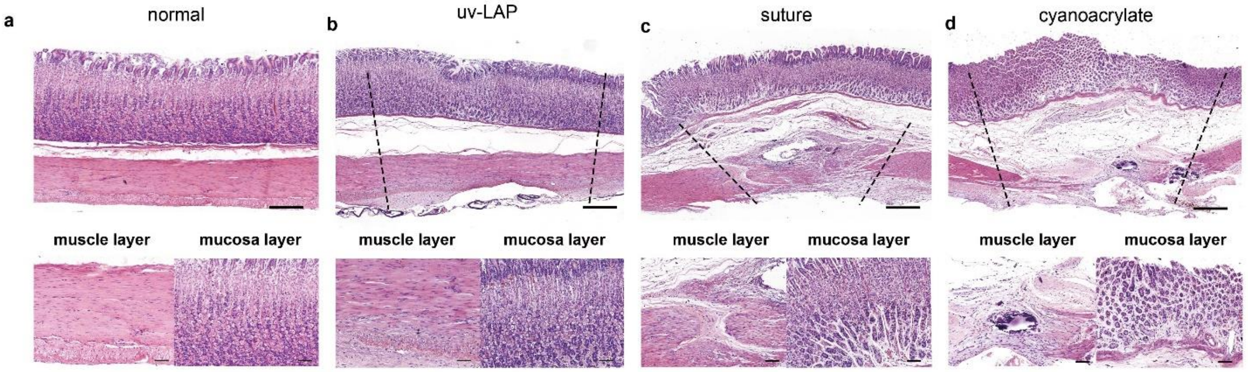

Supplement: Supplementary file 1 — Supporting Information [file ADVS-9-2106115-s001.pdf]
